# Potential of Using Twitter to Recruit Cancer Survivors and Their Willingness to Participate in Nutrition Research and Web-Based Interventions: A Cross-Sectional Study

**DOI:** 10.2196/cancer.7850

**Published:** 2019-05-28

**Authors:** Laura Keaver, Aisling McGough, Mengxi Du, Winnie Chang, Virginia Chomitz, Jennifer D Allen, Deanna J Attai, Lisa Gualtieri, Fang Fang Zhang

**Affiliations:** 1 Clinical Health and Nutrition Centre Department of Health and Nutritional Science Institute of Technology Sligo Sligo Ireland; 2 Friedman School of Nutrition Science and Policy Tufts University Boston, MA United States; 3 Department of Public Health and Community Medicine Tufts University School of Medicine Boston, MA United States; 4 Smith College Northampton, MA United States; 5 Department of Community Health Tufts University School of Arts and Sciences Medford, MA United States; 6 Department of Surgery David Geffen School of Medicine University of California Los Angeles Los Ａngeles, CA United States

**Keywords:** social media, nutrition survey, cancer survivors

## Abstract

**Background:**

Social media is rapidly changing how cancer survivors search for and share health information and can potentially serve as a cost-effective channel to reach cancer survivors and invite them to participate in nutrition intervention programs.

**Objective:**

This study aimed to assess the feasibility of using Twitter to recruit cancer survivors for a web-based survey and assess their willingness to complete web-based nutrition surveys, donate biospecimens, and to be contacted about web-based nutrition programs.

**Methods:**

We contacted 301 Twitter accounts of cancer organizations, advocates, and survivors to request assistance promoting a web-based survey among cancer survivors. The survey asked respondents whether they would be willing to complete web-based nutrition or lifestyle surveys, donate biospecimens, and be contacted about web-based nutrition programs. Survey promotion rate was assessed by the percentage of Twitter accounts that tweeted the survey link at least once. Survey response was assessed by the number of survey respondents who answered at least 85% (26/30). We compared the characteristics of cancer survivors who responded to this survey with those who participated in the National Health and Nutrition Examination Survey (NHANES) 1999-2010 and evaluated factors associated with willingness to complete web-based surveys, donate biospecimens, and be contacted to participate in web-based nutrition programs among those who responded to the social media survey.

**Results:**

Over 10 weeks, 113 Twitter account owners and 165 of their followers promoted the survey, and 444 cancer survivors provided complete responses. Two-thirds of respondents indicated that they would be willing to complete web-based nutrition or lifestyle surveys (297/444, 67.0%) and to be contacted to participate in web-based nutrition interventions (294/444, 66.2%). The percentage of respondents willing to donate biospecimens were 59.3% (263/444) for oral swab, 52.1% (231/444) for urine sample, 37.9% (168/444) for blood sample, and 35.6% (158/444) for stool sample. Compared with a nationally representative sample of 1550 cancer survivors in NHANES, those who responded to the social media survey were younger (53.1 years vs 60.8 years; *P*<.001), more likely to be female (93.9% [417/444] vs 58.7% [909/1550]; *P*<.001), non-Hispanic whites (85.4% [379/444] vs 64.0% [992/1550]; *P*<.001), to have completed college or graduate school (30.1 [133/444] vs 19.9% [308/444]; *P*<.001), and to be within 5 years of their initial diagnosis (55.2% [244/444] vs 34.1% [528/1550]; *P*<.001). Survivors younger than 45 years, female, and non-Hispanic whites were more willing to complete web-based nutrition surveys than older (65+ years), male, and racial or ethnic minority survivors. Non-Hispanic whites and breast cancer survivors were more willing to donate biospecimens than those with other race, ethnicity or cancer types.

**Conclusions:**

Twitter could be a feasible approach to recruit cancer survivors into nutrition research and web-based interventions with potentially high yields. Specific efforts are needed to recruit survivors who are older, male, racial and ethnic minorities, and from socioeconomically disadvantaged groups when Twitter is used as a recruitment method.

## Introduction

### Background

Nearly two-thirds of American adults (65%) use social networking sites, with a particular increase among those 65 years and older (35% in 2015, more than tripled since 2010) [1]. The use of social media has shifted from a focus on personal use to almost all domains including health [1]. Cancer survivors are increasingly utilizing social media to obtain and share health-related information among themselves and with health care providers [2,3]. Social media is also becoming a popular tool for cancer survivors and their caregivers to seek support [4,5].

Cancer survivors have substantially reduced quality of life because of physical and psychosocial late effects [6,7] and are at significantly elevated risk of cancer recurrence and premature death [8]. There is clear evidence to support the benefits of optimal nutrition, ranging from relieving symptoms and treatment-related side effects to improving survival and quality of life among cancer survivors [9-14]. Traditional methods of providing nutrition programs to cancer survivors through outpatient oncology clinics face challenges when cancer survivors experience transportation difficulties or scheduling constraints to participate in these programs in person [15]. Nutrition programs delivered through web-based platforms can potentially circumvent these barriers and reach a broader range of cancer survivors in the community [16-20]. For example, Gorman et al utilized a variety of recruitment methods including social media to recruit young adult female cancer survivors into a research study for reproductive health [16]. The authors collaborated with organizations that support and advocate for adolescent and young adult survivors by posting the recruitment advertisements on Facebook and Twitter approximately every 2 months over a 12-month period and subsequently recruited a total of 381 eligible adolescent and young adult survivors [16]. Compared with other recruitment strategies (eg, clinical-based or community-based) that were also utilized by Gorman et al, social media recruitment provided the highest number of enrolled participants [16]. Attai et al surveyed the knowledge level and psychosocial outcomes in breast cancer survivors who were participants of a Twitter support community for breast cancer survivors by posting the survey link on its Twitter, Facebook page, and blog [4]. This method yielded 206 responses after 2 weeks of survey promotion. In addition, a recent meta-analysis [21] of 12 studies that enrolled 7441 participants for social network site interventions revealed not only favorable outcomes in promoting health behavior change such as weight management, physical activity, and smoking cessation but also a high retention rate: 4 [17-20] of the 6 studies reported a retention rate above 80%, and 2 [22,23] reported retention rates between 65% and 75%. Taken together, social media may represent a cost-effective method for health care providers and cancer support groups to reach cancer survivors in the community and invite them to participate in web-based nutrition intervention programs.

### Objectives

The primary purpose of this study was to evaluate the feasibility of using social media such as Twitter to recruit cancer survivors into nutrition research and web-based interventions and to further assess survivors’ willingness to complete nutrition surveys delivered through this medium, donate biospecimens, and be contacted to participate in future web-based nutrition intervention programs. In addition, this study aimed to compare the demographic and cancer-related characteristics between cancer survivors approached using social media and those from a nationally representative survey.

## Methods

### Study Population and Survey Instruments

We administered the Cancer survivors Adherence to Recommendations for healthy Eating (CARE) survey to cancer survivors. Eligible participants were cancer survivors who were 18 years or older and had been told by a doctor or other health professional that they had cancer or a malignancy of any kind. The survey was self-administered online and included 30 questions. A total of 24 questions asked cancer survivors’ demographic and cancer or treatment-related characteristics, lifestyle habits, perceived barriers for healthy eating and physical activity, and sources of seeking nutrition information. Findings for these questions have been submitted for publication elsewhere. This study specifically focused on the 6 questions about survivors’ willingness to complete web-based nutrition and lifestyle surveys (ie, would you be willing to complete other online surveys about diet, exercise, and lifestyle at a later date?), willingness to donate biospecimens such as oral swab, urine, or blood (ie, would you be willing to use an oral swab kit that we mail to you and you mail back to us? Would you be willing to provide a urine sample using a kit that we mail to you and you mail back to us? Would you be willing to provide a blood sample from a full venous draw, similar to the type of blood draw you would receive at your doctor’s office?), and also survivors’ willingness to be further contacted to participate in nutrition interventions (ie, would you be willing to be further contacted to participate in nutrition programs offered online?), with the available responses being yes, no, or maybe. The study was approved by the institutional review board at Tufts Medical Center/Tufts University.

### Strategies for Survey Promotion

We conducted web-based searches to identify cancer organizations, advocates, and survivors that have active presence in 1 major social media platform, Twitter. To reach active Twitter accounts with a cancer focus, we first located Twitter accounts using the search terms “Cancer Survivor(s),” “Cancer Advocate(s),” “Cancer Support,” “Cancer,” and “Cancer Nutrition” in November 2015. We identified the top 50 Twitter accounts under each of these 5 search terms that met the following inclusion criteria: (1) having 500 or more followers for large cancer organizations (eg, the American Cancer Society) or 200 or more followers for smaller cancer advocate/survivor groups and (2) having contact information such as email address. Due to limitations in resources, we chose to target Twitter accounts that can potentially reach a large number of cancer survivors for survey promotion, such as large cancer organizations that tend to have powerful social media platforms to reach cancer survivors in the community. We also included Twitter accounts of smaller cancer advocate/survivor groups that had a certain number of followers. Although arbitrary, the number of followers specified in the inclusion criteria was chosen to target Twitter accounts that could potentially result in high survey yields. Twitter accounts that advertise or sell nutrition products to cancer survivors or were primarily in a language other than English were excluded. Second, we conducted additional searches in December 2015 in collaboration with Symplur to identify additional accounts that were deemed active in Twitter based on Symplur’s Healthcare Social Graph algorithm [24]. The algorithm ranks Twitter accounts based on (1) the ratio of reactions that each account generates compared with the content it shares and (2) the selectiveness of the social network that each account interacts with. For this additional search, the top 100 Twitter accounts using search term “Cancer” in each of the 2 categories—organizations and advocates—were identified in Symplur. Finally, we created a Twitter account for the CARE survey and identified additional accounts that met the study inclusion criteria among the followers of our Twitter account. Twitter accounts that were identified using all 3 search strategies were subsequently merged, and duplicate or ineligible accounts were removed. A list of Twitter accounts was then finalized, and data were extracted on account name, category, cancer type, contact information, country of origin, and number of followers.

### Survey Administration

**Figure 1:**
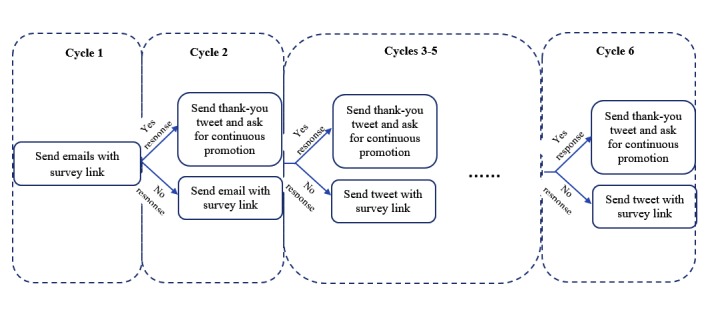
Survey promotion cycles.

A web-based version of the survey was created using SAP Qualtrics survey tools and published with a URL. To administer the CARE survey, we applied 6 arounds (ie, cycles) of contacts to the Twitter accounts identified in the above search (Figure 1). During the first cycle of contact (ie, initial contact), an email was sent to each account. The email included a cover letter that introduced the survey, defined its purpose, and asked the account owner to promote the survey by posting the URL link of the survey on their social media platforms, along with the time frame of survey promotion and sample messages they could post on social media (Textbox 1).

**Sample Twitter messages for survey promotion.**

Sample Twitter messages:

- Cancer Survivors Share Your Thoughts about Nutrition with @TuftsNutrition in @CARE_Study survey link

- Change Eating Habits after Cancer Diagnosis? Tell Scientists @TuftsNutrition in @CARE_Study survey link

In the situation where the email was returned, alternative contact was made through Twitter by sending a tweet with the “@” symbol before the name of the Twitter account. For those who responded to the initial contact by posting the survey link on their social media, a thank you tweet was sent along with a request to continuously promote the survey, by tweeting a link to the survey, until the survey closed. For those who did not respond to the initial contact, a second cycle of contact was made with email or tweet by sending the same cover letter. As tweets were found to generate more responses than emails, after the first cycle, contact was made exclusively by sending tweets that included the survey link. All Twitter accounts were contacted for survey promotion at each cycle even if they had already promoted the survey. The research staff actively followed each account for survey promotion activities at each cycle, for example, tweeting a link to the survey, and recorded in an Excel sheet whether each account tweeted the survey link at least once (yes vs no) per cycle. The number of tweets sent by each account was not recorded. The research staff also monitored survey promotion activities of the followers of the Twitter accounts. The follower accounts were not included in our original list for survey promotion. However, if they promoted the survey by tweeting the survey link, they were subsequently contacted to continuously promote the survey until the survey closed. A total of 6 cycles of contacts were made within about 10 weeks from February 9 to April 23, 2016, and each cycle lasted approximately 1.5 weeks. Respondents who clicked on the survey link were provided with information about the study and asked to provide consent before being able to proceed with the survey. Survey responses completed after each cycle were retrieved from SAP Qualtrics.

### Statistical Analysis

We first described the survey promotion rate achieved at each cycle by calculating the percentage of the Twitter accounts that promoted the survey by tweeting the survey link at least once among those being contacted. We then described the survey response at each promotion cycle by the number of survey respondents who provided complete responses, defined as answering 85% or more of all survey questions. After the survey closed, we exported survey responses from Qualtrics and imported them into SAS 9.4 (SAS Institute) for data checking and cleaning. To assess whether cancer survivors approached using social media such as Twitter differ from cancer survivors in the community in demographic and cancer-related characteristics, we compared cancer survivors who provided complete responses to the CARE survey with those who participated in the 1999-2010 National Health and Nutrition Examination Survey (NHANES), a nationally representative survey that assesses information on health and nutritional status of the noninstitutionalized civilian population in the United States [25]. Continuous variables were compared using analysis of variance, and categorical variables were compared using the chi-square test. Among cancer survivors who provided complete responses to the CARE survey, we further described the percentages of those who indicated that they would be willing to be further contacted for additional nutrition and lifestyle assessments, biospecimen collection, and web-based nutrition interventions. In addition, we evaluated factors associated with willingness to complete web-based nutrition and lifestyle assessments, donate biospecimen, and to be contacted to participate in web-based nutrition programs among survey respondents using logistic regression models adjusted for age, sex, and race/ethnicity. All data analyses were conducted using SAS 9.4.

## Results

### Twitter Accounts

**Figure 2:**
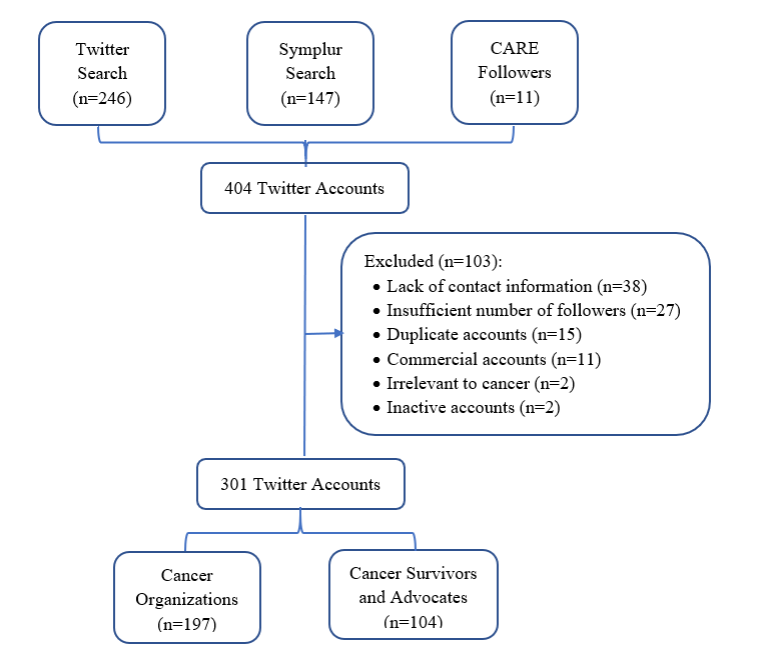
Identification of Twitter accounts for survey promotion. CARE: Cancer survivors Adherence to Recommendations for healthy Eating.

Our initial search identified a total of 404 Twitter accounts, with 246 accounts identified through direct Twitter search, 147 accounts identified through Symplur search, and 11 accounts identified from CARE Twitter followers. Among these accounts, 103 accounts were excluded because of lack of contact information (n=38), the number of followers smaller than the predetermined threshold (n=27), duplicate accounts identified in both Twitter account search and Symplur search (n=15), commercial accounts (n=11), irrelevant to cancer (n=2), and inactive accounts defined as no messages posted in the past 30 days (n=2). The remaining 301 accounts were included in the database for survey promotion at each cycle, including 197 accounts for cancer organizations such as the American Cancer Society and 104 accounts for cancer advocates or survivors such as the Breast Cancer Social Media (#BCSM; Figure 2).

### Survey Promotion and Response Rates

A total of 113 of the 301 accounts (ie, original accounts) promoted the survey over 6 cycles. At each cycle, 28/301 (9.3%), 18/301 (6.0%), 31/301 (10.3%), 21/301 (7.0%), 6/301 (2.0%), and 9/301 (3.0%) promoted the survey, yielding an average promotion rate of 6% per cycle (Table 1). The cancer advocate/survivor accounts yielded a substantially higher average promotion rate (12/104, 11.5%) than cancer organization accounts (6.8/197, 3.5%; *P*<.001). New accounts (n=165) that came from the followers of those originally identified accounts also promoted the survey. The majority of these new accounts were cancer advocates/survivors (133/165, 80.6%) and about one-fifth were Twitter accounts for cancer organizations (32/165, 19.4%). These new accounts were included in the final 4 cycles for survey promotion and the average promotion rate was 20.7% per cycle and 7% (7/99), 19.4% (25/129), 25.9% (38/145), and 26.3% (35/133), respectively, at each cycle (Table 1). There was no significant difference in the average promotion rate of new accounts that were cancer advocates/survivors (23/109, 21.1%) or cancer organizations (3/18, 17%; *P*=.51).

A total of 6 cycles of survey promotion resulted in a total of 584 survey responses, among which 29 respondents identified themselves as not having a cancer diagnosis, and 111 did not provide complete responses (ie, answering at least 85% of the survey questions) and were excluded. Thus, a total of 444/584 (76.0%) cancer survivors provided complete responses to the survey over 10 weeks.

**Table 1:** Survey promotion rates by original and new accounts at each cycle.

Survey cycle		Cancer organization accounts			Cancer advocate or survivor accounts			All accounts		
		Number approached	Number promoted	Promotion rate, %	Number approached	Number promoted	Promotion rate, %	Number approached	Number promoted	Promotion rate, %
**Old accounts**										
	Cycle 1	197	9	4.6	104	19	18.3	301	28	9.3
	Cycle 2	197	12	6.1	104	6	5.8	301	18	6
	Cycle 3	197	11	5.6	104	20	19.2	301	31	10.3
	Cycle 4	197	9	4.6	104	12	11.5	301	21	7.0
	Cycle 5	197	0	0	104	6	5.8	301	6	2
	Cycle 6	197	0	0	104	9	8.7	301	9	3
	Mean per cycle	—^a^	—	3.5	—	—	11.5	—	—	6.3
**New accounts**										
	Cycle 3	9	1	11.1	90	6	6.7	99	7	7.1
	Cycle 4	32	6	18.8	97	19	19.6	129	25	19.4
	Cycle 5	32	6	18.8	115	32	27.8	145	38	25.9
	Cycle 6	0	0	0	133	35	26.3	133	35	26.3
	Mean per cycle	—	—	17.8	—	—	21.1	—	—	20.7

^a^Not applicable.

### Characteristics of Cancer Survivors Approached Using Social Media Versus a National Representative Sample of Cancer Survivors

Compared with a nationally representative sample of 1550 cancer survivors who participated in the NHANES survey, those who responded to the survey promoted using Twitter were significantly younger (53.1 years vs 60.8 years) and more likely to be female (93.9% [417/444] vs 58.7% [909/1550]; *P*<.001), non-Hispanic white (85.4% [379/444] vs 64.0% [992/1550]; *P*<.001), and to have completed college education or higher (30.1% [133/444] vs 19.9% [308/1550]; *P*<.001; Table 2). The majority of survey respondents were from the United States (360/444, 81.1%), with the remaining respondents from Canada (17/444, 3.8%), United Kingdom (13/444, 2.9%), and other countries (54/444, 12.2%).

Breast cancer survivors were the largest survivor group in both surveys, but a substantially higher percentage of breast cancer survivors responded to the social media survey than the national survey (71.2 [316/444] vs 46.2% [716/1550]; *P*<.001). Cancer survivors who responded to the social media survey reported a shorter interval from diagnosis (6.1 years vs 10.5 years; *P*<.001) and were more likely to be within 5 years of their initial diagnosis (55.2% [244/444] vs 34.1% [528/1550]; *P*<.001). In addition, nearly one-third of the respondents to the social media survey were still receiving treatment.

**Table 2:** Characteristics of adult cancer survivors in a social media survey compared with a national sample of cancer survivors.

Characteristics		CARE^a^ (N=444)	NHANES^b^ (N=1550)	*P* value^c^
**Age at survey completion (years), mean (SD)**		53.1 (10.6)	60.8 (14.2)	<.001
	<45, n (%)	97 (21.9)	237 (15.3)	<.001
	45-54.9, n (%)	143 (32.3)	221 (14.3)	—^d^
	55-64.9, n (%)	138 (31.2)	336 (21.7)	—
	65-74.9, n (%)	54 (12.2)	506 (32.7)	—
	≥75, n (%)	11 (2.5)	250 (16.1)	—
**Gender, n (%)**				
	Male	27 (6.1)	641 (33.5)	<.001
	Female	417 (93.9)	909 (58.7)	—
**Race/ethnicity, n (%)**				
	Non-Hispanic white	379 (85.4)	992 (64.0)	<.001
	Non- Hispanic black	13 (2.9)	287 (18.5)	—
	Hispanic	20 (4.5)	226 (14.6)	—
	Other	32 (7.2)	45 (2.9)	—
**Education, n (%)**				
	Grades 0-12	37 (8.4)	837 (54.0)	—
	Some college	120 (27.2)	404 (26.1)	—
	College graduates or above	133 (30.1)	308 (19.9)	<.001
**Primary diagnosis, n (%)**				
	Breast cancer	316 (71.2)	716 (46.2)	<.001
	Other cancer type	128 (28.8)	834 (53.8)	
**Time from diagnosis (years), mean (SD)**		6.1 (6.5)	10.5 (10.6)	<.001
	<5, n (%)	244 (55.2)	528 (34.1)	<.001
	5-9, n (%)	116 (26.2)	385 (24.8)	—
	≥10, n (%)	82 (18.6)	637 (41.4)	—

^a^CARE: Cancer survivors Adherence to Recommendations for healthy Eating.

^b^NHANES: National Health and Nutrition Examination Survey.

^c^For continuous variables (eg, age and time from diagnosis), the *P* values were generated from the analysis of variance (ANOVA) comparing the mean distribution between the 2 groups. For categorical variables (eg, age group, gender, race or ethnicity, education, primary diagnosis, and time from diagnosis group), the *P* values were generated from the Chi-square test comparing the frequency distribution between the 2 groups.

^d^Not applicable.

### Willingness to Participate in Nutrition Research and Interventions

**Figure 3:**
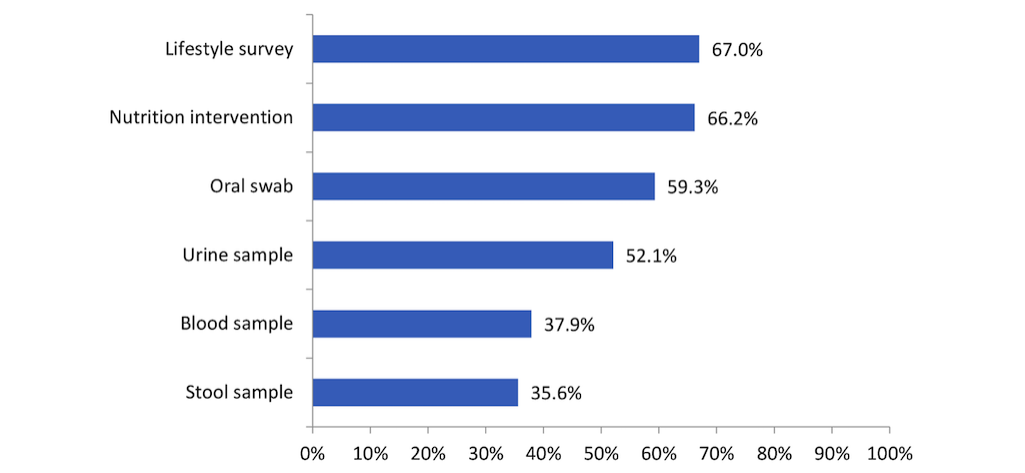
Percentages of the 444 cancer survivors who indicated willingness to complete online nutrition survey, donate biospecimens for research, and willingness to be contacted to participate in online nutrition programs.

About two-thirds (297/444, 67.0%) of the survivors indicated that they would be willing to complete web-based surveys about their nutrition, physical activity, and lifestyle behaviors. The percentages of the cancer survivors who indicated that they would be willing to donate biospecimens were 59.3% (263/444) for oral swab, 52.1% (231/444) for urine sample, 37.9% (168/444) for blood sample, and 35.6% (158/444) for stool sample. About two-thirds (294/444, 66.2%) of the cancer survivors indicated that they would be willing to be contacted further to participate in web-based nutrition intervention programs (Figure 3).

### Factors Associated With Willingness to Complete Online Nutrition Survey, Donate Biospecimen, and to Be Contacted to Participate in Web-Based Nutrition Programs

Survivors’ willingness to complete web-based nutrition surveys, donate biospecimens, and be contacted to participate in future nutrition programs through web-based platforms did not differ by survivors’ demographic and cancer-related characteristics with a few exceptions: survivors who were 65 years or older were less willing to complete web-based nutrition surveys compared with survivors who were younger than 45 years (odds ratio, OR=0.4, 95% CI 0.2-0.8); female survivors were more willing to complete web-based nutrition surveys than male survivors (OR=2.8, 95% CI 1.2-6.6); and survivors who had race other than non-Hispanic white were less willing to complete surveys (OR=0.6, 95% CI 0.3-1.0) or donate biospecimens (OR=0.4, 95% CI 0.2-0.7) compared with non-Hispanic white survivors, whereas breast cancer survivors were more willing to donate biospecimens than survivors of other cancer types (OR=1.7, 95% CI 1.0-2.8; Table 3).

**Table 3:** Factors associated with willingness to complete web-based lifestyle survey, donate biospecimen, and to be contacted to participate in web-based nutrition programs online among adult cancer survivors.

Variable		Willingness to complete web-based nutrition survey, OR^a^ (95% CI)^b^	Willingness to donate biospecimen, OR (95% CI)^b^	Willingness to be contacted to participate in web-based nutrition programs, OR (95% CI)^b^
**Age at survey completion (years)**				
	<45	Ref^a^	Ref	Ref
	45-54.9	0.9 (0.5-1.6)	0.9 (0.5 – 1.6)	1.0 (0.6-1.8)
	55-64.9	1.4 (0.8-2.5)	1.3 (0.8 – 2.4)	1.1 (0.6-2.0)
	≥65	0.4 (0.2-0.8)	0.6 (0.3 – 1.1)	0.6 (0.3-1.2)
**Gender**				
	Male	Ref	Ref	Ref
	Female	2.8 (1.2-6.6)	1.7 (0.7 – 3.9)	1.9 (0.8-4.4)
**Race/ethnicity**				
	Non-Hispanic white	Ref	Ref	Ref
	Other	0.6 (0.3-1.0)	0.4 (0. 2- 0.7)	0.8 (0.4-1.4)
**Education**				
	Grades 0-12	Ref	Ref	Ref
	High school/some college	1.5 (0.9 – 2.6)	1.0 (0.4 – 2.4)	0.6 (0.3 – 1.6)
	College graduate or higher	1.2 (0.7 – 2.1)	1.0 (0.4 – 2.2)	1.2 (0.5 – 2.7)
**Body mass index (kg/m^2^)**				
	<25	Ref	Ref	Ref
	25-29.9	0.4 (0.3 – 0.7)	1.0 (0.6 – 1.6)	1.1 (0.7 – 1.8)
	≥30	0.3 (0.2 – 0.5)	1.5 (0.9 – 2.6)	1.2 (0.7 – 2.0)
**Primary diagnosis**				
	Other	Ref	Ref	Ref
	Breast cancer	1.5 (0.9 – 2.5)	1.7 (1.0 – 2.8)	1.6 (1.0 – 2.7)
**Treatment status**				
	On-treatment	Ref	Ref	Ref
	Off-treatment	1.0 (0.6 – 1.6)	1.0 (0.6 – 1.5)	1.0 (0.7 – 1.6)
**Time from diagnosis (years)**				
	<5	Ref	Ref	Ref
	05-11	1.0 (0.6 – 1.7)	0.8 (0.5 – 1.3)	1.0 (0.6 – 1.6)
	≥10	0.8 (0.5 – 1.5)	1.1 (0.6 – 2.0)	1.3 (0.7 – 2.3)

^a^OR: odds ratios.

^b^Odds ratios and 95% CIs were adjusted for age, sex, and race/ethnicity.

^c^Ref: reference.

## Discussion

### Principal Findings

Our study is among the first that utilizes Twitter as an exclusive method to recruit cancer survivors for web-based survey that assessed survivors’ willingness to participate in nutrition research and to be contacted to participate in future web-based interventions. Our results suggest that Twitter is a feasible approach to reach cancer survivors in the community and supports the potential of delivering web-based nutrition interventions to this population.

Using a systematic approach, we identified a list of Twitter accounts of both large cancer organizations and smaller cancer advocate and survivor groups to promote the survey. Although the average promotion rate among the original accounts was low, the total yield for survey responses was still promising: a total of 584 individuals responded to the survey, and 444 cancer survivors provided completed responses over 10 weeks. Interestingly, the Twitter accounts that were not originally included in the contact list (ie, new accounts) had a much higher promotion rate, which may reflect the chain referral effect of snowball sampling associated with social media promotion. Despite the survey spanning over 10 weeks, the majority (74.1%) of our survey responses were received during the first cycle of survey promotion (ie, the initial 1.5 weeks), and fewer survey responses were received beyond the first 3 cycles of survey promotion. Thus, the initial 1 to 3 cycles (ie, the first 5 weeks) of the survey promotion is likely to result in the highest yield. These findings may represent the unique characteristics of survey promotion using Twitter.

### Comparison With Prior Work

Similar to cancer survivors who responded to a social media survey reported by Attai et al [4], cancer survivors who responded to this Twitter survey tended to be young, female, non-Hispanic white, and receive a high level of education. As such, specific efforts are needed to enhance the representativeness of cancer survivors in a social media survey by reaching those who are older, male, and from racial/ethnic minorities or socioeconomically disadvantaged groups. Although social media recruitment was particularly effective in reaching breast cancer survivors, additional efforts are required to recruit cancer survivors with other cancer diagnoses that tend to be under-represented using social media recruitment. Future research should look to determine why this medium poses a challenge for recruitment of these particular groups, such as potential barriers in accessing or using social media and differences in motivations for participating in nutrition-related research [26,27]. Recruiting through social media groups of specific cancer types or reaching socioeconomic disadvantaged groups through community-based organizations may be combined with general social media recruitment to improve the representativeness of the population. Over half of the cancer survivors in our sample were within 5 years of their initial cancer diagnosis and nearly one-third were still receiving cancer treatment. This contrasts the finding that the majority of the cancer survivors in the general population who participated in NHANES were long-term survivors (ie, ≥10 years post diagnosis). These findings suggest that cancer survivors who are recently diagnosed might be more responsive to social media recruitment than long-term survivors.

Nearly two-thirds of the cancer survivors who responded to our survey reported that they were willing to participate in future nutrition research and to be contacted about future interventions. This finding supports the feasibility of utilizing Twitter to recruit cancer survivors for intervention and to employ it as a tool to deliver the intervention. Although social media holds great promise as a means of delivering health promotion, its use in the context of cancer research is still in its infancy. Few studies have utilized social media as a channel to deliver lifestyle interventions to cancer survivors [2]. One study that delivered educational materials and messages to promote physical activity within closed Facebook groups reported a significantly greater increase in light physical activity (135 min/week) and weight loss (2.1 kg) over 12 weeks among 86 young adult cancer survivors [23]. The fact that the intervention was delivered entirely using Facebook and a self-monitoring site is promising and supports the feasibility of utilizing social media or other online platforms to deliver interventions to cancer survivors at a lower cost with a broader reach. Studies are needed to further evaluate how to leverage social media to promote health behaviors in cancer survivors and whether the behavioral change can be sustained. More broadly, research is needed to understand how social media is changing health communication in cancer care and to evaluate the possibility of incorporating social media into cancer care to provide optimal nutrition support [2,28].

### Limitations

Our study has limitations. Although we developed a systematic approach to identify influential social media accounts for survey promotion, the number of followers we used to determine influential Twitter accounts is arbitrary. There are no standard or accepted methods to rate the influence of social media accounts. When identifying influential Twitter accounts through Symplur search, we adopted the ranking algorithm of the Symplur that provides specific assessments on Twitter accounts’ active presence in health care. However, there have been few evaluations on Symplur’s ranking algorithm; and it is possible that we failed to include other Twitter accounts that have an impactful social media platform to reach cancer survivors in the community. Second, we did not intend to identify accounts from other social media platforms such as Facebook. As Twitter accounts may be more heavily used by younger individuals, whereas Facebook can potentially reach more diverse groups, our findings may not be generalized to other social media recruitment methods [28].

### Conclusions

In summary, the use of Twitter could be a promising approach to recruit cancer survivors in the community into nutrition research and interventions. About two-thirds of the cancer survivors reached through Twitter were willing to complete web-based nutrition and lifestyle surveys, donate biospecimens, and to be contacted to participate in future web-based nutrition programs. However, cancer survivors who responded to this social media recruitment tended to be younger, female, non-Hispanic white, and have a high level of education and were skewed to breast cancer survivors. Future research is warranted to identify effective approaches to reach a diverse and representative sample of cancer survivors using social media and to evaluate the cost-effectiveness of adapting nutrition interventions for web-based or social media delivery to improve the nutritional intake and long-term health of cancer survivors in the community.

## Abbreviations

CARE: Cancer survivors Adherence to Recommendations for healthy Eating
NHANES: National Health and Nutrition Examination Survey
OR: odds ratio
